# Oral health educational interventions for pharmacists and pharmacy staff: A scoping review

**DOI:** 10.1016/j.rcsop.2025.100658

**Published:** 2025-09-14

**Authors:** Hayley Man, Ajesh George, Arash Rudman, Meng-Wong Taing, Angela Masoe, Leanne Smith, Woosung Sohn, Bradley Christian

**Affiliations:** a Sydney School of Public Health, Faculty of Medicine and Health, The University of Sydney, Sydney, New South Wales 2050, Australia; b Western Sydney Local Health District, NSW Health, Westmead, New South Wales 2145, Australia; c Sydney Dental School, Faculty of Medicine and Health, The University of Sydney, Sydney, New South Wales 2050, Australia; d Australian Centre for Integration of Oral Health, School of Nursing and Midwifery, Western Sydney University, New South Wales, Penrith 2751, Australia; e Ingham Institute for Applied Medical Research, Liverpool, New South Wales 2170, Australia; f School of Nursing, Faculty of Science, Medicine and Health, University of Wollongong, Wollongong, NSW 2500, Australia; g School of Pharmacy, The University of Queensland, Brisbane, Queensland 4072, Australia; h Centre for Oral Health Strategy, NSW Ministry of Health, St Leonards, New South Wales 2065, Australia

**Keywords:** Education, Oral health, Pharmacists, Pharmacy staff, Professional development, Training

## Abstract

**Introduction:**

Oral diseases are a significant public health issue globally, however timely access to healthcare can assist in reducing this disease burden. Pharmacists and pharmacy staff are increasingly being recognised as a valuable health service resource, especially in rural-remote areas where access to dental services is limited. Access to oral health training is a challenge that has been identified to enable pharmacists and staff to integrate oral healthcare into their routine practice.

**Aim:**

To identify and examine the characteristics of existing oral health education interventions for pharmacy staff including evaluation outcomes of the identified interventions.

**Method:**

Medline, Embase and CINAHL databases were searched. Citation searching and a structured grey literature search was performed using search engine Google, OAIster, BASE, dental and pharmacy organization websites. The review method was informed by the Preferred Reporting Items for Systematic Reviews and Meta-Analyses extension for Scoping Reviews (the PRISMA-ScR). Studies were eligible if they described an educational intervention focused on oral health for pharmacists or pharmacy staff, in any setting, and reported characteristics, content, delivery, or evaluation outcomes.

**Findings:**

Ten oral health educational interventions for pharmacists and pharmacy staff were identified. The most common topics covered were general oral health promotion and management of common oral presentations in pharmacies. Most interventions had a single delivery format, such as a standalone online module or printed resource, rather than a suite of complementary materials combining multiple formats. Four were accredited as continuing professional development. Three identified interventions required membership to professional organisations. One resource had published literature on an evaluation process which reported that participants considered the content acceptable, relevant, and feasible to incorporate into pharmacy practice; however, it did not measure changes in knowledge, attitudes, confidence, or practice quantitatively.

**Conclusions:**

There are very limited publicly accessible oral health educational interventions for pharmacy staff, and little evidence on their impact including KAP (Knowledge Attitudes and Practices), confidence, feasibility and acceptability. Addressing these gaps could support pharmacists to play a greater role in meeting oral health needs, particularly in underserved areas.

## 1 Introduction

Oral diseases are a significant public health issue and among the most prevalent non-communicable diseases globally, affecting an estimated 3.5 billion people - approximately half of the world's population across low-, middle- and high-income countries.^1^ Untreated dental caries (tooth decay) is the most prevalent and widespread disease affecting an estimated two-billion people, with a global prevalence of 43 % in the primary dentition and 29 % in the permanent dentition.^2^^,^^3^

In Australia, dental caries is the most common oral disease in children, with 40 % experiencing decay in their primary teeth and 32 % of adults have untreated dental caries.^4^^,^^5^ Despite this high prevalence only 49 % of adults and 57 % of children under the age of five visited a dental practitioner in the past 12 months.^5^ Poor oral health impacts on systemic health as well as a person's quality of life, and is associated with cardiovascular disease, pneumonia, diabetes mellitus and low birth weight.^6^ Dental pain can disrupt eating, speaking, sleeping, socialising, and negatively affecting growth and development. Oral diseases also contribute to hospital admissions,^7^ school absenteeism, reduce work productivity, and economic costs for families and society.^8^

Multiple barriers contribute to delayed treatment and inequitable access to care. These include financial costs, long public dental waitlists and eligibility criteria.^9^ People living in rural and remote areas face additional challenges, including a shortage of dental practitioners (due to recruitment and retention issues), limited dental facilities, increased treatment costs and reduced transport options.10., 11., 12 Rural and remote communities are also more likely to lack fluoridated water and face higher costs for healthy food and oral hygiene products.^12^^,^^13^ Poor oral health literacy – often linked with chronic disease and limited oral health promotion in general health settings is another barrier.^14^, ^15^, ^16^

The burden of disease and the limitations of the current treatment-focused model of care highlight the need for alternative, cost-effective, and sustainable approaches. One proposed strategy is the integration of oral health into primary care. The Global Oral Health Action Plan (GOHAP) 2023–2030 recognises the need to expand workforce models and integrate oral health into primary health services.^17^ Strategic Objective 3 focuses on competency-based education among health workers and Strategic Objective 4 targets integration of oral health into primary health services, with a goal that by 2030, 80 % of countries will deliver oral health services through primary health facilities.^17^ This review aligns with GOHAP Actions 45, 48 and 62, which highlight involving pharmacists in oral health care, promoting equitable access to oral health education and integrating oral health care into primary care.^17^

The Global Oral Health Action Plan is also supported by recent findings in a systematic review that explored the integration of oral health care into care. It recognised that non-dental personnel can contribute to improved access to oral health care, but the right policies, incentives and training need to be available.^18^ Various integration strategies were identified with the majority relating to the delivery of oral healthcare by non-dental primary care professionals through training, education and/or policy changes.^18^ In Australia, several successful integration strategies involving non-dental professionals demonstrate feasibility and cost effectiveness. The Early Childhood Oral Health (ECOH) program enables child health professionals to identify children at risk of dental caries and make timely referrals.^19^ The Midwifery Initiated Oral Health (MIOH) program trains midwives to screen, educate, and refer women for dental care during antenatal visits, improving maternal oral health and increasing dental attendance.^20^ In general practice, targeted education and collaborative models have supported the integration of oral health into chronic disease management plans, facilitating early detection and referral of at-risk patients^18^ These initiatives demonstrate that cost-effective, policy-supported integration is possible in Australia and could serve as a framework for expanding integration into other primary care settings.

Health professionals, public health practitioners and academics have advocated for community pharmacies to play a greater role in oral health promotion and disease prevention.^21^, ^22^, ^23^ Studies across multiple countries have shown that pharmacy staff are frequently consulted regarding oral health concerns.^21^, ^22^, ^23^, ^24^, ^25^

Despite this progress, little is known about how oral health integration could be implemented in community pharmacy. Pharmacies are easily and frequently accessed by the general population,^26^ play a role in local community, particularly in rural and remote areas^27^ and are well placed to contribute to oral health promotion and prevention strategies.^28^, ^29^, ^30^ However, for pharmacy staff to fulfill their role as a potential source for oral health information, evidence-based training and interventions should be provided. This will address the key challenges for pharmacy staff to provide oral health advice which include lack of interventions, knowledge and confidence around promoting oral health; with limited formal training relating to oral health.^30^, ^31^, ^32^, ^33^

Our preliminary literature search suggested that few oral health educational interventions for pharmacy staff exist and that formal training opportunities are scarce. The extent, nature and characteristics of these interventions have not been systematically mapped. A scoping review was therefore warranted to identify and describe existing oral health education interventions for pharmacy staff, examine the evaluation outcomes, and consolidate the evidence base. This will help confirm and clarify knowledge gaps, inform policy development, and inform the development of evidence-based and effective oral health educational interventions for community pharmacy practice.

## 2 Aim

This scoping review aimed to systematically identify and describe existing oral health educational interventions for community pharmacy staff, including their characteristics and any reported evaluation outcomes, to inform policy development and the design of evidence-based training programs.

## 3 Methods

### 3.1 Study design

A scoping review was conducted to present a broad overview of the evidence currently available in this emerging area, to identify knowledge gaps, to inform future research agendas and policy development.^34^ A scoping review allowed the team to consider a broad range of grey literature including clinical guidelines and tools, reports, and consensus statements. The review was guided by the Joanna Briggs Institute (JBI) methodology and its 2020 update, following the framework outlined by Peters et al.^34^^,^^35^ The scoping review was reported according to the Preferred Reporting Items for Systematic reviews and Meta-Analysis (PRISMA) extension for Scoping Reviews (PRISMA-ScR) guidelines.^36^

### 3.2 Search strategy and selection criteria

An electronic search of Medline (Ovid, 1946 to 2024), Embase (Ovid, 1947 to 2024) and CINAHL (inception to 2024) were conducted. The review question was formulated using the Population, Concept, Context (PCC) framework as recommended by the Joanna Briggs Institute for scoping reviews which guided the development of inclusion criteria and mapping of key search terms (Table 1).^37^ The search strategy for each database was developed with the assistance of an experienced librarian and incorporated both controlled vocabulary (MeSH, CINAHL Headings, and Emtree) and free text keywords relevant to oral health, pharmacy, and education. Boolean operators (AND/OR) and truncations were applied as appropriate, and strategies were applied to each database. Full search strategies for each database are provided in Appendix 1 to enhance transparency and reproducibility. Backward and forward searches using the reference list of articles were also performed.

**Table 1:** Search Terms by PCC framework.

Population	Pharmacist Pharmacy Pharmacy staff Pharmacy assistant Pharmacy technician Chemist
Concept: Oral Health	Dental health Oral health Dental disease Oral disease Mucosal disease Oral healthcare
Concept: Education	Training Professional development Educational resource Health education Inservice training Intervention Instruction Knowledge Scope
Context	Global – all countries considered

The grey literature search was structured and comprehensive^38^. It included hand searching of relevant primary sources including organization websites from English-speaking countries. Defined combinations of search terms were applied to search engines including Google (screening the first 200 results) and to open-source archives including OAIster and the Bielefeld Academic Search Engine (BASE). Further manual searching was undertaken across websites of relevant dental and pharmacy organisations in English-speaking countries. In addition, a global call for data was disseminated via social media platforms, including Twitter and LinkedIn, as well as through public health, dental, and pharmacy professional channels, inviting submission of unpublished studies, program reports, and other grey literature. Measures were taken to ensure balanced international coverage and reduce potential bias towards Australian resources. Duplicates from grey literature sources were removed and records were managed in the same way as those retrieved from electronic databases.

EndNote and Covidence was used to manage a two-stage screening process. Two reviewers (HM and BC) independently conducted the screening of titles and abstracts, followed by full-text eligibility assessment, using Covidence software. Discrepancies were resolved by consensus discussion.

### 3.3 Eligibility criteria

Resources were included if they targeted community pharmacists or community pharmacy staff and involved oral health education interventions aimed at improving KAP (Knowledge Attitudes and Practices) as well as outcomes including confidence, feasibility, and acceptability. These outcomes were considered separately but complementary to KAP and informed inclusion. Included educational interventions compromised but not limited to: fact sheets, screening tools, online learning modules, in person training and booklets. Studies from all publication dates and countries were eligible.

Studies not published in English or studies lacking educational interventions were excluded.

### 3.4 Data extraction and synthesis

Covidence software was used to manage the data extraction synthesis. Data extracted included resource type, target audience, oral health topics covered, delivery format, accreditation status and evaluation outcomes such as measures of knowledge, attitudes, practices, confidence, feasibility and acceptability, resource characteristics and evaluation.^39^ One reviewer extracted data into an evidence table, and a second reviewer verified a proportion of the data for accuracy. Refinement and clarification of extracted data were conducted through team discussion.

A narrative synthesis approach was chosen to systematically and comprehensively assess the results and highlight key findings without bias. To minimise bias during synthesis, a predefined data extraction framework was used, and data verification by two independent reviewers was conducted. Resources for which only publicly available information was found, and no formal evaluation data were available, were not subjected to further assessment. Results were tabulated, and organised to explore similarities, differences, and patterns between identified educational resources.^39^

## 4 Results

### 4.1 Literature search and data extraction

**Fig. 1:**
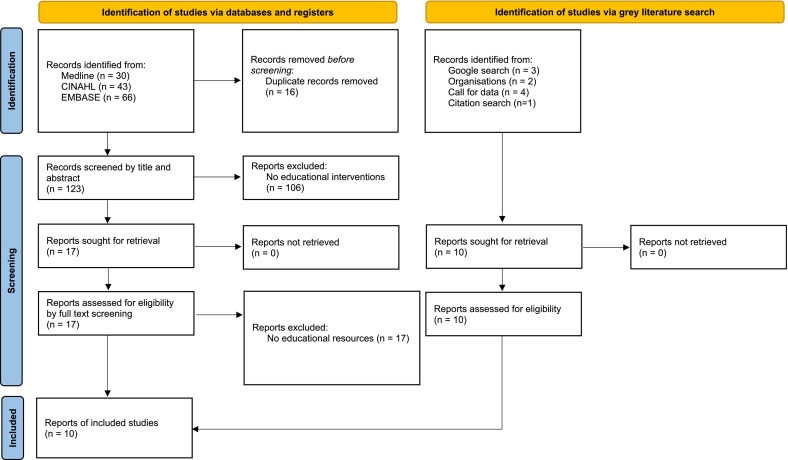
*Study selection process: PRISMA.*^35^

Electronic searches yielded 139 results (Fig. 1). After title and abstract screening in Covidence, 17 scientific papers were identified as potentially eligible but all were excluded at full-text review as they did not include educational interventions. The grey literature search identified three interventions through Google, two through organisational websites, four through the call for data and one through citation searching (Fig. 1).

A total of ten oral health educational interventions for community pharmacists and pharmacy staff met the inclusion criteria. Their key characteristics including author/organization, location, type, target audience, topic/theme, characteristics and evaluation. The characteristics analysed included content, format, delivery and duration. Evaluation components included reported information related to knowledge, attitudes, practices (KAP), confidence, feasibility and acceptability, though formal evaluations were limited. The data is presented in Table 2.

**Table 2:** Reported Evaluation and Available Data on Knowledge, Attitudes, Practices, Confidence, Feasibility, and Acceptability.

Author/Organization Location	Target audience	Education Type	Characteristics: Contents, format and delivery, duration Accessibility Endorsement/Accreditation by professional organization	Evaluation: KAP⁎, confidence, feasibility, acceptability.
The Pharmacy Guild of Australia^40^ Australia	Pharmacists	Online course	Topics: Maintaining oral health. Insight into oral health, focusing on periodontal health. Duration: 0.5 h. Accessible with paid membership. Accredited by the Australian Pharmacy Council for Continuing Professional Development (CPD).	No evaluation based on publicly available information
National Health Service (NHS)^46^ England	Community pharmacists	Flow chart (single page) and fact sheets	Topics: Oral health and oral pain management in children and young people Dental abscess, pain, and mouth ulcers. Flow chart: health promotion and referrals Fact sheets: definitions, tips, photos, links to additional resources.	Audit conducted at end of campaign to establish learning and development needs. Results not publicly available.
Dental Health Services Victoria^41^ Australia	Pharmacists and pharmacy teams	Fact sheet (2 pages)	Topics: Pharmacy teams supporting oral health. Key messages for good oral health, oral disease risk factors, referrals. Freely available to download from website. Endorsed – Victorian State Government, Healthy Families Healthy Smiles, Pharmaceutical Society of Australia (Vic), Dental Health Services Victoria and Australian Dental Association (Vic Branch).	No evaluation based on publicly available information.
International Pharmaceuticals Federation (FIP)^49^ Netherlands	Pharmacists	Handbook	Topics: Periodontal disease as a diabetes complication. Diabetes prevention, screening, and management	No evaluation based on publicly available information.
Health Education England (HEE) Pharmacy London^45^ England	Community pharmacists	15 Fact sheets (single page)	Topics: Common dental and oral health presentations to community pharmacies. Bleeding and swollen gums, \ post op extraction, chipped teeth, oral hygiene advice, access information, dry mouth, fractured dental appliances, lost fillings/ crowns, oral fungal infections, myofascial pain), ulcers(wisdom tooth), teething, toothache. Factsheets: Prompts for pharmacists. Freely accessed (online or printable format). Endorsed by the National Health Service (NHS).	Evaluation – pre-evaluation tally chart and online evaluation links. At time of access in October 2023, online evaluation link not functional, hence evaluation areas could not be confirmed.
SA Dental Service^42^ Australia	Pharmacists	Booklet/patient resources and online training module.	Topics: Pharmacies as a setting for oral health promotion. Oral health care advice, oral health information resources, medications and oral health, oral health products, oral health screening and referral.	Evaluation – number of pharmacies distributed, number of clicks/views, number of people accessed training.
American Association of Colleges of Pharmacy (AACP)^48^ USA	Pharmacists	8 Online courses	Topics: Integration of oral health and primary care. Oral to systemic health, oral health(adult/child/women/geriatric), acute dental problems, caries risk assessment, Duration: 1 h each. Freely accessible information following website registration. Accredited by the Accreditation Council for Pharmacy Education for Continuing Professional Education	No evaluation based on publicly available information.
The County Durham and Darlington Foundation Trust (CDDFT) oral health promotion team^44^ England	Pharmacists, pharmacy technicians and pharmacy assistants	In person training session (Pilot program)	Presentation topics: caries prevention, fluoridated toothpaste, and dietary advice. Practical session topics with phantom demonstrator head: oral hygiene instructions and denture care.	Post qualitative evaluation was performed. A patient questionnaire and semi-structured qualitative interviews with pharmacy staff were conducted.
Pharmaceutical Society of Australia (PSA)^43^ Australia	Pharmacists	3 online modules	Topics: Common oral health presentations to pharmacies. Teething pain, Dry mouth management Duration: 0.75 h Accessible with organisational membership/payment by non-members. Accredited by the Australian Pharmacy Council for Continuing Professional Development (CPD).	No evaluation based on publicly available information.
Centre for pharmacy postgraduate education (CPPE)^47^ England	Pharmacists, pharmacy technicians working in community pharmacies	*E*-learning program with workbook.	Topics: Urgent care: the role of the community pharmacy and the NHS CPCS scheme. Dental pain and tooth decay covered in subsection of common conditions in the pharmacy. Duration: 2 h Booklet freely accessible online. E-learning module requires payment or professional education funding by NHS England. Endorsed by Health Education England NHS and The University of Manchester.	

⁎ *Abbreviation:* *KAP (Knowledge Attitudes and Practices).*

### 4.2 Location/target audience

All interventions gathered were from English-speaking developed countries. Four were from Australia,40., 41., 42, 43. four from the United Kingdom,^44^, 45., 46, 47. one from the United States^48^ and one from the Netherlands.^49^ All interventions reported pharmacists as the target audience, three specifically targeted community pharmacists45., 46, 47. and three included all pharmacy staff as the target audience.^41^^,^^44^^,^^47^

### 4.3 Topic/theme

For two interventions, oral health was included as a subchapter of the broader topic; diabetes^48^ and urgent care.^47^ The remainder of the interventions specifically covered oral health topics. Four interventions provided information to community pharmacies on how to manage common oral and dental presentations.^43^^,^45., 46, 47. One resource specifically focused on managing common oral and dental presentations in children and young people.^46^ Four interventions covered general oral health promotion and hygiene,^40^^,^^41^^,^^44^^,^^48^ however the specific content of some of these interventions was not clear as they required organisational membership to access detailed information. Only one resource^48^ clearly indicated that it covered a broad range of oral health topics, including oral health across different age groups, acute dental problems as well as oral to systemic health. It appeared to be the only educational resource that covered the association between oral and systemic health.

### 4.4 Format/endorsement

Educational interventions were available in different formats. Four were online courses,^40^^,^^43^^,^^48^^,^^47^^–^ three were fact sheets^41^^,^^45^^,^^46^ and three included a handbook/booklet.^42^^,^^47^^,^^49^ Only two included interventions in more than one format,^42^^,^^47^ however one of these was not exclusively covering oral health.^47^

Two required payment or membership to the organisational website.^40^^,^^43^ It is important to note that paid membership to these professional organisations is not compulsory for local practicing pharmacists. Hence, these interventions are not accessible to all pharmacists. One resource appeared to be freely accessible only to pharmacists working or contracted to the government health body.^47^ While the remainder of interventions did not appear to have professional membership or paywall restrictions, it is important to note that only three of these were easily found through Google without a call to data through professional channels.

Four interventions were accredited for continuing professional development by official pharmacy bodies.^40^^,^^43^^,^^47^^,^^48^ Five others were endorsed by or associated with a professional organization. One did not include any endorsements or professional association.^42^ Of the interventions that could be freely accessed, three offered practical tips and tools,^44^, 45., 46 and four explained how to refer to local oral health services.^42^^,^^44^, 45., 46

### 4.5 Evaluation

Only one resource had published literature on an evaluation process,^44^ which included qualitative data from a pilot oral health promotion program involving community pharmacies. This evaluation reported positive feedback regarding the acceptability of community pharmacies as locations for oral health interventions and a perceived positive change in knowledge and attitude towards oral health. Pharmacy staff provided positive feedback for the program as well and identified the need for further oral health education. The effectiveness of the intervention in improving the knowledge and confidence of pharmacy staff and improved oral health outcomes for patients were not explored.

Two interventions presented as a booklet with online training^42^ and a series of fact sheets^45^ reported either an ongoing or potentially completed evaluation process, but the methodology and results have not been published or publicly available. One of these interventions indicated an audit process to collect data after implementation looking to establish learning and development needs of the pharmacists involved.^45^ One resource^42^ collected quantitative data on frequency of resource access, but again methodology and results have not been published. Overall, formal evaluations assessing KAP (Knowledge, Attitudes, and Practices), confidence, feasibility, and acceptability were limited or unavailable for most resources.

## 5 Discussion

This is the first review to identify oral health educational interventions for community pharmacists and pharmacy staff, and it is evident that there are limited interventions in this area and even less that have been evaluated.

Of the limited interventions identified, most focused on general oral hygiene promotion or management of common presentations to community pharmacies. Interventions in the format of fact sheets provided practical pointers rather than in depth knowledge and theory on the topics.^41^^,^^45^^,^^46^ The online modules, by their descriptions, appeared to provide more in-depth knowledge around their relevant topics, however they required payment or organisational membership to confirm their content. Successful oral health programs for non-dental health professionals that have shown improvements in use of dental services, patients' oral health knowledge and outcomes; have included a suite of training interventions and tools.^50^ This aligns with findings from other countries, such as the United Kingdom and Canada, where broader primary care–based oral health programs have successfully combined online and in-person learning, clinical tools, and referral pathways to improve service integration.^18^ This contrasts with the interventions identified in this review which were mostly in one format, rather than in depth training courses with accompanying tools to guide and assist practice. There is room for more comprehensive and complementary oral health learning and training interventions to be developed for pharmacists and pharmacy staff across the lifespan, since they are a key health care provider with regular contact with many people.

Similar challenges have been reported in educational interventions targeting pharmacists in other health areas such as smoking cessation, asthma management, mental health, and antimicrobial stewardship, where multi-modal, accredited, and accessible training programs have demonstrated more robust outcomes.^51^, ^52^, ^53^, 54. Adopting these evidence-based models, particularly those that integrate accreditation, practical tools, and follow-up support, could strengthen oral health education for pharmacists internationally.

The International Pharmaceutical Federation (FIP) provides global frameworks and competency-based development goals for pharmacy education, which include competencies relevant to health promotion and disease prevention, including oral health.^54^ Similarly, the World Health Organization (WHO) promotes the integration of oral health into primary care and highlights the role of pharmacists in health promotion.^17^^,^^55^, 56. The WHO's “Seven-Star Pharmacist” concept describes pharmacists as caregivers, decision-makers, communicators, managers, life-long learners, teachers, and leaders.^56^ These roles require key knowledge, attitudes, and practices (KAP) for oral health promotion — such as clinical competence, effective patient communication, public health advocacy, and teamwork with other healthcare professionals. The educational interventions identified in this review, although limited in scope and accessibility, have the potential to strengthen several of these competencies — particularly knowledge and communication skills — thereby supporting pharmacists to fulfill aspects of the Seven-Star model in the context of oral healthcare. However, the lack of robust evaluation means the extent of improvement in these KAP remains unclear, highlighting a priority area for future research.

Our findings indicate that while these frameworks recognise pharmacists as key health providers in preventive health, existing oral health educational interventions for pharmacists are limited in number, format, and accessibility. This gap suggests that current practice falls short of fully realising FIP's competency goals and WHO's strategic objectives, underscoring the need for targeted, accessible, and accredited oral health training aligned with these global standards.

Only four of the interventions were officially accredited as professional development by official pharmacy bodies, suggesting there is limited evaluation of the quality of the educational interventions available. In addition to quality control, accreditation of courses and training as continuing professional development (CPD) can also serve as a motivation to increase the uptake of educational programs by pharmacists. This review suggests that there needs to be more robust evaluation of oral health interventions for pharmacists and pharmacy staff to ensure an evidence-informed approach to resource development. For oral health educational interventions and interventions to have positive and sustained outcomes in the pharmacy setting based on previous successful programs, it is important to have high quality evaluation during the development and implementation through randomized controlled trials^50^ or pilot studies^51^ that are sufficiently powered to see an effect. As only one study looked at short term qualitative outcomes, there is very limited information on whether existing oral health programs for pharmacists are effective at influencing the quality and quantity of oral health care provided in the pharmacy setting. Evaluations should assess whether they firstly improve knowledge and confidence, and secondly whether they contribute to improvement in oral health outcomes. In addition, evaluations should also study the process of implementing pharmacy oral health models of care to identify optimal implementation strategies. Internationally, similar evaluation principles have been applied in other health workforce training initiatives recommended by the World Health Organization (WHO) and International Pharmaceutical Federation (FIP), offering potential frameworks for oral health program assessment.

The data gathered from this review confirms that there are not only very limited training interventions in oral health available for pharmacists and pharmacy staff, but there is also very limited information on the quality and outcomes of training. This review has also highlighted that interventions are not easily and readily found or accessible. The limited access to educational interventions, particularly those requiring paid membership to organisations or programs, acts as a barrier for pharmacists and pharmacy staff to access training. Even freely available interventions were difficult to find without relevant professional contacts. This is in keeping with previous studies where Australian pharmacy staff identified lack of knowledge/ongoing training and interventions to assist practice as key barriers to providing guidance and counselling to oral health presentations. This is despite more than half of pharmacy staff having some form of oral health engagement in their practice.^32^ Comparable barriers have been reported in rural and remote pharmacy settings in other countries, highlighting the need for globally coordinated strategies to improve availability and discoverability of training resources. This gap is a challenge that needs to be addressed to allow for the increased and effective integration of oral healthcare into the pharmacy setting.

### 5.1 Strength

A structured grey literature search as well as data gathering through professional contacts was conducted. This reduced publication bias and allowed interventions that did not have a peer reviewed evaluation process to be identified.

### 5.2 Limitations

Although a structured grey literature search was performed, it is possible that not all relevant interventions were identified. For example, interventions missed could include non-English interventions, interventions located on private intranets, and interventions with restricted access by organisational or professional membership. Limited information was obtainable from a number of pharmacy and professional organisations due to paywall restrictions to access educational interventions and content. However, several Australian interventions were accessed through professional contacts of the authors, skewing the location of interventions found. There could be many more such training interventions hidden behind professional organisations that did not come up in the search, despite a global call for information.

For the interventions identified, data extraction was performed on the information that was publicly accessible. This review did not include reaching out to the developers of the interventions directly, which could have potentially yielded more detailed information on the characteristics and particularly on any evaluation process that was conducted and data that was collected. Future research should include direct engagement with developers and professional bodies, both nationally and internationally, to access more complete program descriptions and evaluation data.

Although the majority of interventions identified in this review were not formally evaluated, available evidence from the included studies and from other health domains suggests that well-designed educational interventions can improve pharmacists' knowledge, confidence, and practice behaviours, and in turn enhance patient outcomes. For example, in smoking cessation and asthma management, pharmacist-delivered interventions have been associated with measurable improvements in patient health indicators and service utilisation.^51^, ^52^, ^53^, 54. Similar approaches in oral health — particularly those incorporating interactive training, practical tools, and follow-up support — could be expected to improve oral health literacy and facilitate earlier referral, thereby benefiting both pharmacists and the communities they serve. However, further research is required to confirm these effects in oral health contexts.

### 5.3 Future direction and research implications

This scoping review has provided valuable information to inform the topic of pharmacy and oral healthcare. The lack of published scientific literature on the topic is a major evidence gap but not surprising given that interest in this area has gained momentum only recently. The review also showed that there is very little publicly available, educational interventions on the topic of oral health in the pharmacy setting. Evaluation of educational interventions was a key area that needs to be strengthened in the future. Addressing these research gaps will lead to the development of evidenced based oral health models of care involving pharmacists and pharmacy staff that could be implemented across various countries and settings. Such integrated models of care have the potential for major impact on oral health especially in rural-remote areas that face the double whammy of poor access to dental services and increased disease burden.

Specific recommendations include developing multi-format, accessible, and accredited oral health education programs that combine theoretical knowledge with practical tools such as screening checklists and referral pathways. Oral health education should also be integrated into existing pharmacy CPD frameworks and undergraduate curricula, aligned with global standards from FIP and WHO. Ensuring equitable access by addressing barriers such as paywalls and membership restrictions is essential, as is fostering international collaboration to adapt training materials, establish joint accreditation, and strengthen the global evidence base.

## 6 Conclusion

This review showed that there is a shortage of oral health educational interventions for pharmacy staff to support their role in oral health promotion. Variances were noted among the characteristics including the accessibility, delivery and topics discussed. In addition, the evaluation outcomes in terms of knowledge, practices, confidence, feasibility and acceptability appears poorly explored and difficult to access.

## Clinical relevance

Oral diseases are a worldwide public health issue. Oral diseases can negatively impact a person's quality of life and require timely access to oral health care for effective management. Involving pharmacists and pharmacy staff in integrated health models of oral health care may assist in timely access and reduced disease burden. However, a key component of such integrated models is the requirement to provide context-specific evidence-informed oral health education and training, to ensure that appropriate oral health care can be provided by pharmacists and pharmacy staff in the pharmacy setting.

## CRediT authorship contribution statement

**Hayley Man:** Writing – review & editing, Writing – original draft, Methodology, Formal analysis, Data curation, Conceptualization. **Ajesh George:** Writing – review & editing, Supervision, Methodology, Conceptualization. **Arash Rudman:** Writing – review & editing, Methodology. **Meng-Wong Taing:** Writing – review & editing, Methodology. **Angela Masoe:** Writing – review & editing, Methodology. **Leanne Smith:** Writing – review & editing, Project administration. **Woosung Sohn:** Writing – review & editing, Methodology. **Bradley Christian:** Writing – review & editing, Supervision, Methodology, Formal analysis, Data curation, Conceptualization.

## Declaration of competing interest

The authors declare that they have no competing interests.

## Data Availability

The authors confirm that the data supporting the findings of this study are available within the article and/or its supplementary materials. The protocol was not registered.

## Appendix A Supplementary data

Supplementary material 1 Image 1

Supplementary material 2 Image 2

Supplementary material 3 Image 3
