# Supplementary material for: Oral health educational interventions for pharmacists and pharmacy staff: A scoping review
Source: Explor Res Clin Soc Pharm. 2025 Sep 14;20:100658. doi: 10.1016/j.rcsop.2025.100658 (PMC12494839; doi:10.1016/j.rcsop.2025.100658)
Supplement: Supplementary file 1 — Supplementary material 1 [file mmc1.docx]

Appendix 1 search strategy

**1. Medline (Ovid) Search Strategy**

*(MeSH terms are in CAPS with [MeSH] label)*

1. **exp Oral Health/** [MeSH: *Oral Health*]
2. oral health*.tw.
3. dental health*.tw.
4. oral disease*.tw.
5. dental disease*.tw.
6. 1 or 2 or 3 or 4 or 5
7. **health education/** [MeSH: *Health Education*] OR **health education, dental/** [MeSH: *Dental Health Education*]
8. education* resource*.tw.
9. **exp education, dental/** [MeSH: *Dental Education*] OR **exp inservice training/** [MeSH: *Inservice Training*]
10. (professional* adj3 develop*).tw.
11. training.tw.
12. 7 or 8 or 9 or 10 or 11
13. **Pharmacists/** [MeSH: *Pharmacists*]
14. pharmacy staff.tw.
15. pharmacy assistant*.tw.
16. pharmacy technician*.tw.
17. 13 or 14 or 15 or 16
18. 6 and 12 and 17

**CINAHL Search Strategy**

*(CINAHL Headings are in CAPS with [CINAHL] label)*

1. **MH "Oral Health"** [CINAHL]
2. TI oral health* OR AB oral health*
3. TI dental health* OR AB dental health*
4. TI oral disease* OR AB oral disease*
5. TI dental disease* OR AB dental disease*
6. S1 OR S2 OR S3 OR S4 OR S5
7. **MH "Health Education+"** [CINAHL] OR **MH "Dental Health Education"** [CINAHL]
8. TI education* resource* OR AB education* resource*
9. **MH "Dental Education+"** [CINAHL] OR **MH "Staff Development"** [CINAHL] OR **MH "Inservice Training"** [CINAHL]
10. TI professional* N3 develop* OR AB professional* N3 develop*
11. TI training OR AB training
12. S7 OR S8 OR S9 OR S10 OR S11
13. **MH "Pharmacists"** [CINAHL]
14. TI pharmacy staff OR AB pharmacy staff
15. TI pharmacy assistant* OR AB pharmacy assistant*
16. TI pharmacy technician* OR AB pharmacy technician*
17. S13 OR S14 OR S15 OR S16
18. S6 AND S12 AND S17

**Embase (Ovid) Search Strategy**

*(Emtree terms are in CAPS with [Emtree] label)*

1. **exp oral health/** [Emtree: *oral health*]
2. oral health*.ti,ab.
3. dental health*.ti,ab.
4. oral disease*.ti,ab.
5. dental disease*.ti,ab.
6. 1 or 2 or 3 or 4 or 5
7. **exp health education/** [Emtree: *health education*] OR **dental health education/** [Emtree: *dental health education*]
8. education* resource*.ti,ab.
9. **dental education/** [Emtree: *dental education*] OR **inservice training/** [Emtree: *inservice training*]
10. (professional* adj3 develop*).ti,ab.
11. training.ti,ab.
12. 7 or 8 or 9 or 10 or 11
13. **exp pharmacist/** [Emtree: *pharmacist*]
14. pharmacy staff.ti,ab.
15. pharmacy assistant*.ti,ab.
16. pharmacy technician*.ti,ab.
17. 13 or 14 or 15 or 16
18. 6 and 12 and 17
