# Supplementary material for: Oral health educational interventions for pharmacists and pharmacy staff: A scoping review
Source: Explor Res Clin Soc Pharm. 2025 Sep 14;20:100658. doi: 10.1016/j.rcsop.2025.100658 (PMC12494839; doi:10.1016/j.rcsop.2025.100658)
Supplement: Supplementary file 3 — Supplementary material 3 [file mmc3.docx]

***Figure 2*** *Grey literature identification and selection process [37]*

| **Step 1: Identification of Search Terms**  Key terms were identified using the PCC framework:  - Oral health, dental health, pharmacy - Education, training, professional development  **Step 2: Systematic search of peer-reviewed literature**  Conducted in commercial databases to identify indexed grey literature or supplementary resources  **Step 3: Hand searching of primary sources**  Manual search of professional and organisational websites in English-speaking countries: - Australian Dental Association - Pharmaceutical Society of Australia - Pharmacy Guild of Australia - Royal Pharmaceutical Society (UK) - General Pharmaceutical Council (UK) - American Pharmacists Association - International Pharmaceutical Federation (FIP) - WHO Oral Health Unit  **Step 4: Web-based grey literature search**  Application of search terms to: - Google (first 200 results per search) - OAIster - BASE (Bielefeld Academic Search Engine) - Call for data: Twitter, LinkedIn, professional networks |
| --- |
